# The functional regulatory details of ERK2 in complex with RSK1: an *in silico* insight†

**DOI:** 10.1039/d1ra01020d

**Published:** 2021-03-16

**Authors:** Sepideh Jafari, Farzaneh Mohamadi Farsani, Maziar Ganji, Mohamad Reza Ganjalikhany

**Affiliations:** Department of Cell and Molecular Biology, Faculty of Biological Science and Technology, University of Isfahan Isfahan Iran m.ganjalikhany@sci.ui.ac.ir +98-31-37932250 +98-31-37932250; Department of Medical Genetics, School of Medicine, Shahid Beheshti University of Medical Sciences Tehran Iran

## Abstract

Protein kinases play a significant role in cellular activation procedures by exhibiting a vivid selection in the target, as well as recognizing and phosphorylating them. Extracellular signal-regulated kinase 2 (ERK2) is one of the main kinases in the mitogen-activated protein kinase (MAPK) signaling cascade and engages in dynamically regulating the activities of signaling proteins and physiological processes, including cell proliferation, differentiation, adhesion, migration, and survival. Predicting collective dynamic and structural motions in biological macromolecules is pivotal to obtain a better understanding of the majority of biological processes. Here, through molecular dynamic simulation and normal mode analysis, we investigated ERK2 conformations, in the forms of active (phosphorylated), inactive (unphosphorylated), and in a complex with its substrate, ribosomal protein S6 kinase alpha-1 (RSK1), to determine functional characteristics. Our finding demonstrated that ERK2 plays a switch role in the regulation of pathways. In the case that this protein kinase is in the active form, all critical regions shift to be prepared to accept the substrate and catalytic action. Meanwhile, inactive ERK2 shows contrasting results in which all motions tend to close the catalytic site and cease the phosphorylation action in the MAPK cascade. These findings are in line with those from other similar studies and provide us with novel molecular target regions and recent details on how this mechanism works.

## Abstract 2

DFG, αC-helix orientation regarding the active site position and distance between K54 and Glu71 in the active and inactive states of ERK2.

## Graphical abstract

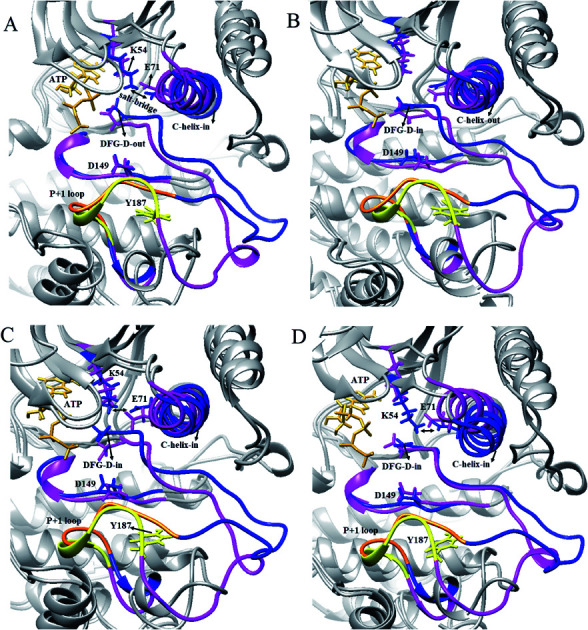

## 1. Introduction

Mitogen-activated protein kinase (MAPK) pathways regulate diverse functions in cells. These pathways are activated by various stimuli, continue into the nucleus, and phosphorylate numerous proteins affecting gene expression, metabolism, division, and survival of the cell. Dysregulation of MAPK cascades leads to cancer and other human diseases.^1,2^ There are four different MAPK families, including extracellular signal-regulated kinases 1 and 2 (ERK1/2), extracellular signal-regulated kinases (ERK5), c-Jun N-terminal kinase (JNK), and p38 MAPK (p38). The RAS/RAF/MEK/ERK cascade is an important MAPK signaling pathway and includes a small G protein (RAS) and three protein kinases (RAF, MEK, ERK). RAS protein could be phosphorylated through the binding of various growth factors to the cell surface receptors (receptor tyrosine kinase, RTK).^3,4^ Activated RAS recruits and activates RAF, a serine/threonine-protein kinase. Phosphorylated-RAF then activates MEK1/2 (MAPK/ERK kinase), dual-specificity protein kinase, and activated MEK1/2 phosphorylates ERK1/2. Finally, activated-ERK1/2 affects several substrates and different transcription factors, which would alter the gene expression, whether in cytosol or nucleus.^5,6^

ERK1/2 are expressed in most tissues in mammals. Human ERK1 and ERK2 are 84% identical in sequence, and share many, but not all, functions. ERK1/2 contains unique N- and C-terminal extensions that lead to their signaling specificity. There is a 17-residue insertion in the N-terminal of ERK1 that is absent in ERK2. There is also a 31-residue insertion in the ERK1/2 family, called kinase insert domain (KIM), which causes additional specificity for these proteins.^7^ Human ERK1 and ERK2 consist of 379 and 360 residues, respectively, and like other kinases have a glycine-rich loop, catalytic loop, activation loop, P + 1 loop, D-domain, and F-domain. In the development of mice, the *erk1* gene is dispensable; nonetheless, the ablation of the *erk2* gene results in embryonic death. Due to the higher expression levels of ERK2, it has been studied more than ERK1.^8,9^ There are 160 target molecules downstream of ERK1/2, such as different transcription factors (Ets-1, c-Jun, c-Myc, and NF-κB), cytosolic phospholipase A2, ternary complex factor (TCF) family, cytoskeletal proteins, apoptotic proteins, and ribosomal S6 kinase (RSK) family. RSK, a protein-serine/threonine kinase with influential roles in cell regulation, is one of the cytosolic substrates of ERK1/2. Growth factors, polypeptide hormones, neurotransmitters, and chemokines induce the activation of the RSK pathway.^10^ The only available crystal structure for ERK1/2 in the complex with its substrates is ERK2–RSK1 (ribosomal protein S6 kinase alpha-1) with PDB ID 4NIF^11,12^ (Fig. S1†). The RSK family enzymes are composed of an N-terminal kinase domain (NTKD), a C-terminal kinase domain (CTKD), and 100 amino acids as a linker between them. RSK interacts with ERK1/2 through two interfaces, linear motif (interface 1) and Thr573, which will be phosphorylated by the active site of ERK2 (Asp149) (interface 2). The RSK family has various cytoplasmic and nuclear targets that can be alluded to as transcription factors (TIF1A, CREB, serum response factor, NF-κB), several ribosome-associated proteins related to the protein synthesis (eIF4B and ribosomal protein S6), and cell-survival signals (Bcl2).^13^ The dysregulated expression of RSK causes several human diseases comprising brain injury, cancer, cardiac hypertrophy, diabetes, and inflammation. An up-regulated RSK signaling pathway is observed in several types of cancers such as colon cancer. This pathway, moreover, mediates tumor invasion and metastasis.^14–17^

In this study, *in silico* methods, including molecular dynamics simulation and normal mode analysis have been performed on three forms of ERK2 including active-ERK2, inactive-ERK2, and ERK2 in a complex with RSK1 in order to deeply understand and explain the functional details behind the structure and dynamics of ERK2. It is noteworthy that the motions of each one of these three forms were precisely examined and compared with each other. To prove the results, normal mode analysis was performed by calculating the lowest-frequency modes with a high degree of collectivity.

## 2. Materials and methods

### 2.1 Preparation of structures

Crystal structures of ERK2 were obtained from Protein Data Bank in active (2ERK), inactive (3SA0), and ERK2–RSK complex (4NIF) forms for molecular dynamics (MD) simulations. Structural investigations were performed by Swiss-PDB viewer 4.10 and Chimera1.12.^18,19^ VMD 1.9.1 has also been used to visualize ERK2's structural dynamics during MD simulations.^20^ All the structures have been depicted in ribbon and surface representations in order to provide a clear and simplified image of the proteins without considering all-atom details.

### 2.2 Molecular dynamics simulations

Molecular dynamics simulation was performed using AMBER 14 with ff14SB force field.^21,22^ The structures were solvated in a truncated octahedron box with a TIP3P water model in a 10 Å hydration layer. Structural neutralization was done by adding Na^+^ ions to the structures (3 Na^+^/12 493 H_2_O for inactive-ERK2, 6 Na^+^/12 024 H_2_O for active-ERK2, and 2 Na^+^/17 817 H_2_O for the complex), and then the coordination and topology files were saved for the next steps of MD simulations.

Minimization of ERK2 with ions and water was performed in two steps. First, water and ion and then the whole system were minimized with 4000 steps (2500 steps of steepest descent, followed by 1500 steps of the conjugate gradient). Non-bonded interactions were calculated at a cutoff distance of 10 Å by PME (Particle Mesh Ewald) method in periodic boundary conditions.^23^

The system was heated from 0 to 300 K for 150 ps, using Langevin thermostat in NPT ensemble.^24^ Then, the equilibration was done for 600 ps in the NPT ensemble. Finally, production MDs were performed in 300 ns for all forms with the NPT ensemble. Additionally, the SHAKE algorithm was used for hydrogen atoms. The coordinates were then saved every 20 ps. All of the simulations were repeated three times, each for 300 ns with random initial velocities.

### 2.3 Analysis of trajectories

In order to analyze the MD production, *cpptraj* from AMBER Tools 15 was used.^22^ The root mean square deviation (RMSD), root mean square fluctuation (RMSF), and the radius of gyration (Rad_gyr_) were calculated regarding the initial structure (eqn (S1)–(S3)†). The distances between key regions were calculated to compare the active, inactive, and complex forms of ERK2 with the initial structure during the simulation. In order to identify important residues and motifs that are related to the catalytic reaction, the accessible surface area was studied.

### 2.4 Normal mode analysis

The normal mode analysis of ERK2 was performed by Elastic Network Model (*ElNemo*) server.^25^ Lowest-frequency modes (modes 7–12) with a high degree of collectivity were calculated. Distance fluctuation maps of residues and mean square displacement were depicted as a measure of structural flexibility.

## 3. Results

### 3.1 Molecular dynamics studies

**Fig. 1:**
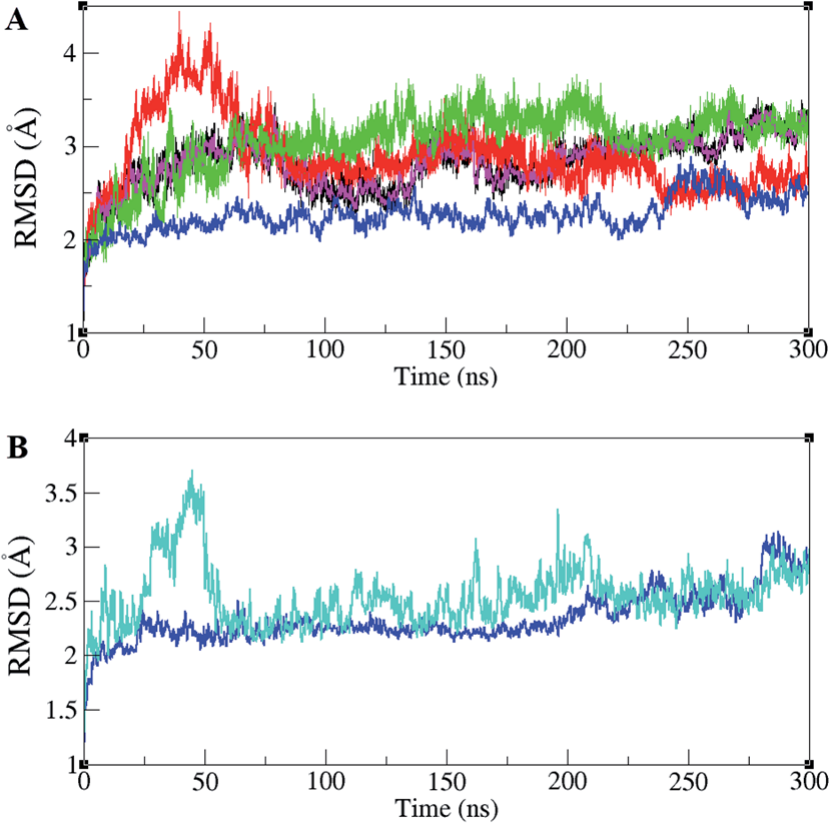
Root mean square deviation (RMSD) in ERK2 and ERK2–RSK1 complex during the simulations. (A) RMSD values for inactive-ERK2 with ATP–Mg^2+^ (pink), inactive-ERK2 without ATP–Mg^2+^ (red), active-ppERK2 with ATP–Mg^2+^ (green), active-ppERK2 without ATP–Mg^2+^ (blue). The blue graph shows the active-ERK2 with phosphorylated Thr185 and Tyr187 and in the absence of ATP–Mg^2+^. An increase in the RMSD value in red graph (inactive-ERK2 without ATP–Mg^2+^) is clearly visible in the beginning of the simulation. (B) RMSD values for ERK2 (blue) and RSK1 (turquoise).

ERK1/2 are activated by upstream proteins such as RAF, RAS, and MEK. Active-ERK2, also, affects downstream proteins. RSK1 is one of the ERK2's substrates that is activated after the ERK2–RSK1 association. This activated RSK1 is transferred to the nucleus and phosphorylates transcription factors. Based on dynamic changes in ERK2 protein kinase, the active (PDB ID 2ERK), inactive (PDB ID 3SA0), and complex states with RSK1 (PDB ID 4NIF), have been simulated. RMSD values of different forms of ERK2 during simulation are illustrated in Fig. 1A. As shown in Fig. 1A, active-ERK2 with phosphorylated Thr185 and Tyr187 (ppERK2), in the absence of ATP–Mg^2+^ (blue graph), was more stable in comparison with other structures. An increase in the RMSD value in inactive-ERK2, without ATP–Mg^2+^ (red graph), was vividly visible at the beginning of the simulation, which is due to the conformational changes of the structure. The RMSD of ERK2–RSK1 (performed in 300 ns) is also shown in Fig. 1B. As depicted in Fig. 1B, it seems that RSK1 has undergone structural rearrangements at the beginning of the simulation, but ERK2 showed more stability during the simulation and the RMSD value increased slightly by the end of the simulation.

**Fig. 2:**
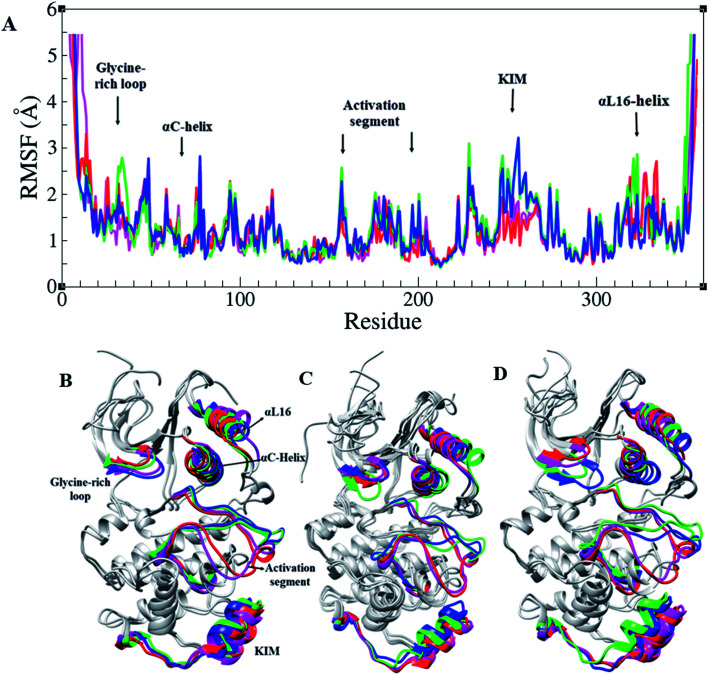
Root mean square fluctuation (RMSF) in ERK2. (A) RMSF graphs and (B–D) 3D structures of ERK2 in different forms of ERK2 colored based on their functional segments. Inactive-ERK2 with ATP–Mg^2+^ (pink), inactive-ERK2 without ATP–Mg^2+^ (red), active-ppERK2 with ATP–Mg^2+^ (green) and active-ppERK2 without ATP–Mg^2+^ (blue).

RMSF of Cα atoms in different forms of ERK2, active, inactive, and in the complex form with RSK1, is shown in Fig. 2A. Based on RMSF graphs, glycine-rich loop,^32–37^ αC-helix (61–74), catalytic loop (147–154), activation segment (DFG motif, activation loop, and APE motif (167–197)), and KIM (a 31-residue sequence insertion within the kinase domain (248–277)), which are functionally important, represented various levels of flexibility. The active forms of ERK2 (blue and green) and the inactive forms (pink and red) had a different flexibility pattern during the simulations. In order to activate the MAPK pathway, ERK2 interacts and phosphorylates RSK1, such that Thr573 from RSK1 was phosphorylated by Asp149 in the catalytic loop of ERK2 and these residues had a low RMSF value (Fig. 2 and S1†).

According to the *R*_gyr_ graphs, the active forms of ERK2 (green and blue) had the lowest Rad_gyr_ during the simulation (Fig. S2†). The active-ERK2 in the presence of ATP–Mg^2+^ (green) revealed the tightest packing owing to the movement of the C-lobe and N-lobe in a closer orientation in the structure. On the other hand, inactive forms of ERK2 (red and pink) had lower compactness. In the ERK2–RSK1 complex, the value of Rad_gyr_ for RSK1 fluctuates between 21.5 to 22.5 Å, meanwhile, Rad_gyr_ for ERK2 is more stable and has a small variation around 21.5 Å.

The activation segment has important roles in both substrate binding and the catalytic activity of protein kinases. The activation segment in most of the kinases begins with a DFG motif (Asp167–Phe168–Gly169 in ERK2) and activation lip (Thr185, Glu186 and Tyr187). The P + 1 loop (^187^YVATRWYR^194^) is located in the middle and ends with an APE motif (Ala195, Pro196 and Glu197).^7^ All MAP kinases only phosphorylate substrates with proline in the P + 1 site.^26–28^

In order to study the differences between the active and inactive forms of ERK2, the activation segment was examined during the simulation. The results clarified that the conformation of the activation segment in the active ERK2 was entirely different from the inactive structure. As shown in Fig. 2, in the active-ERK2 forms (blue and green), the activation segment tended to move towards the αC-helix, whereas, in the inactive-ERK2, it tended to conceal the P + 1 loop, which is responsible for recognizing the substrate.

**Fig. 3:**
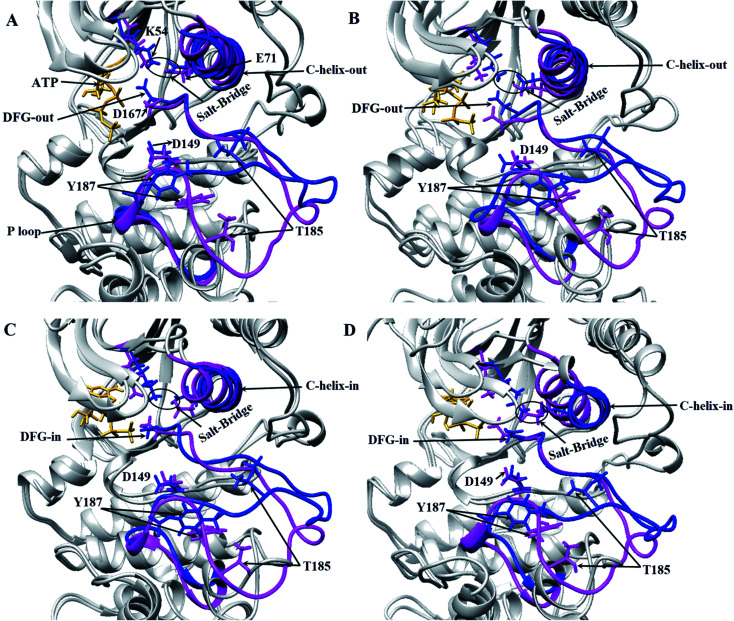
3D representations of ERK2 at different simulation frames. Critical areas in ERK2, including the orientation of DFG and αC-helix regarding the active site position, the distance between Lys54 (K54) and Glu71 (E71) in active and inactive states of ERK2 in different frames during the simulation have been depicted. The inactive-ERK2 and active-ERK2 are shown in pink and blue, respectively. Structures were obtained from four different frames based on RMSD graph value: (A) are the starting frames for active form, the DFG and αC-helix are in DFG-out and αC-helix-out state. In the inactive form, DFG-in and αC-helix-out were observed and salt-bridge in both states exists. (B) In the middle of the simulation for active-ERK2, DFG-out, αC-helix-out and salt-bridge were observed. In inactive-ERK2, DFG-in, αC-helix-out were observed and salt-bridge does not exist. (C) In the middle of the simulation, the DFG-in, αC-helix-out in both forms were observed and salt-bridge is still stable in the active-ERK2. (D) At the end of simulation for active-ERK2, DFG-in, αC-helix-in were observed and K54 and E71 are in close contact, while in inactive-ERK2, αC-helix-out, DFG-in were observed and salt-bridge does not exist.

Three important residues in protein kinases were Lys, Asp and Asp in the K/D/D motif with catalytic activity. In ERK2, Lys54 in the β3 N-lobe, Asp149 in the catalytic loop (plays the catalytic role) and Asp167 in DFG motif (binds to Mg^2+^ and coordinates the ATP) were the components of the K/D/D motif. Fig. 3 and S3† show the distances between the Lys/Asp/Asp (K/D/D) motif components during the simulation. As depicted, the distances between Lys54–Asp149 and Lys54–Asp167 in the active-ERK2 had the lowest values. Meanwhile, the distance between Asp149 and Asp167 was increased in the active form due to the placement of ATP and its interaction with these residues. In order to determine the roles of the motifs related to the catalytic reaction, the characterization of ERK2's accessible surface area was performed. Based on accessible surface area calculations, the surface area of Lys54, Asp149, and Asp167 in the active-ERK2 was less accessible than the inactive forms (Fig. S4†). In the active form, Lys54 involves with Glu71 in a salt bridge and Asp149 interacts with OH of Ser/Thr/Tyr kinase (such as Thr573 from RSK1) in order to prepare it for phosphorylation and, eventually, Asp167 is involved in the interaction with Mg^2+^ and coordination of ATP (Fig. 3 and S5†).

The DFG motif in the activation segment of ERK2 has different conformations in active and inactive forms. In the active-ERK2, the aspartate side chain of DFG (Asp167) moves towards the active site called the “DFG-aspartate-in” (binds to Mg^2+^ and coordinates the charges of phosphates in ATP), whereas in the inactive state it moves away from the active site (“DFG-aspartate-out”).^29^ These fluctuations are also observed in active and inactive ERK2. As illustrated in Fig. 3, the DFG conformation, the in active-ERK2, was in DFG-out conformation until the middle of the simulation and then turned to DFG-in, but this process was in the opposite way for the inactive-ERK2 as the conformation was in DFG-in in the beginning and then turned to DFG-out by the end for the simulation.

Another important and conserved region is αC-helix (residues 61–74), which is located in the N-lobe of ERK2. The αC-helix in active-ERK2 faces into the active site, known as “αC-helix-in”, while its orientation is altered to “αC-helix-out” in inactive-ERK2. It is noteworthy that ERK2 is active in both “αC-helix-in” and “DFG-in”. Besides, Glu71 in the αC-helix is connected to Lys54 (in β3 N-lobe), which is considered a salt-bridge. In active-ERK2, the salt-bridge was established and the connecting residues were getting closer to each other during the simulation, meanwhile, in inactive-ERK2, these two residues were getting far from each other. In other words, the kinase was inactive in the absence of the salt-bridge.^7^ As shown in Fig. 3C and D, in the active-ERK2, the distance between Lys54 and Glu71 was decreased and the salt-bridge was established during the simulation.

In order to evaluate the integrity of the ERK2–RSK1 complex, the distances between Lys54–Glu71 that are involved in the salt bridge in ERK2, as well as Asp149 in ERK2 and Thr573 in RSK1, have been calculated (Asp149 is involved in the interaction with Thr573 to prepare RSK1 for phosphorylation). As represented in Fig. S6,† the distance between Lys54–Glu71 and Asp149–Thr573 was increased gradually until the middle of the simulation and then came back to the initial point by the end of the simulation.

**Fig. 4:**
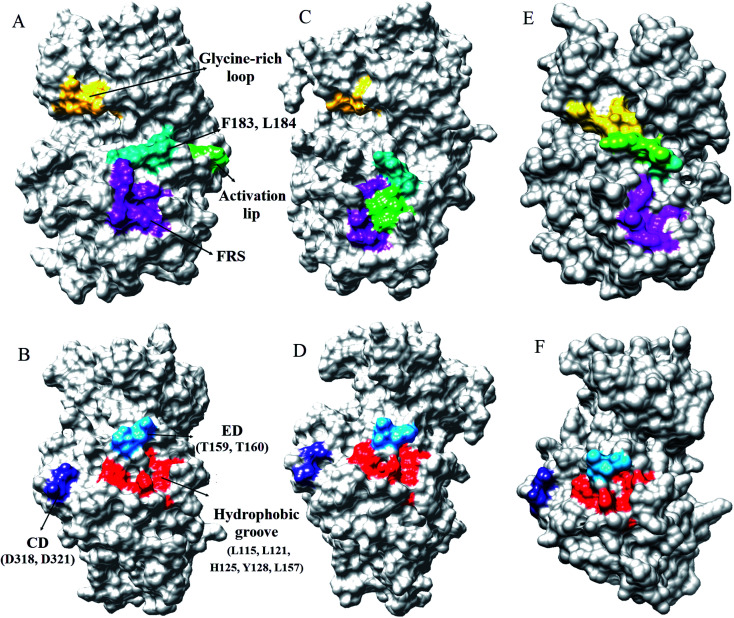
The accessible surface areas of DRS, FRS, glycine-rich loop, and activation loop in two states of ERK2. (A and B) Active-ERK2, (C and D) inactive-ERK2, and (E and F) ERK2–RSK1 complex. The accessible surface areas of these residues were higher in active form than in the inactive form.

Every substrate possessed one or two docking domains for interaction with a specific kinase. The D-site and F-site are two docking domains that help ERK1/2 to interact with up/down-stream proteins. DRS, D-recruitment site (including L121, H125, Y128, L157, T159, T160, D318, and D321) and FRS, F-recruitment site, (including Leu200, Tyr233, Leu234, Leu237, and Tyr263) in ERK2 were related to the D-site and F-site docking domains, respectively. The accessible surface areas of DRS and FRS are shown during the simulation in Fig. 4 and S7–S9.† As it is illustrated, the accessible surface areas of these residues were higher in the active form than in the inactive form. As shown in Fig. 4, in the active state of ERK2, the distance between these residues is lower than in the inactive form. Similar to the active-ppERK2, these residues are closer to each other for the ERK2–RSK1 complex. Based on the results, active-ERK2 has a more compact structure than inactive-ERK2. Finally, the complex form had the most compact structure. The activation segment and the glycine-rich loop in the active-ppERK2 had the most significant conformational changes during the simulation.

### 3.2 Normal mode analysis

**Fig. 5:**
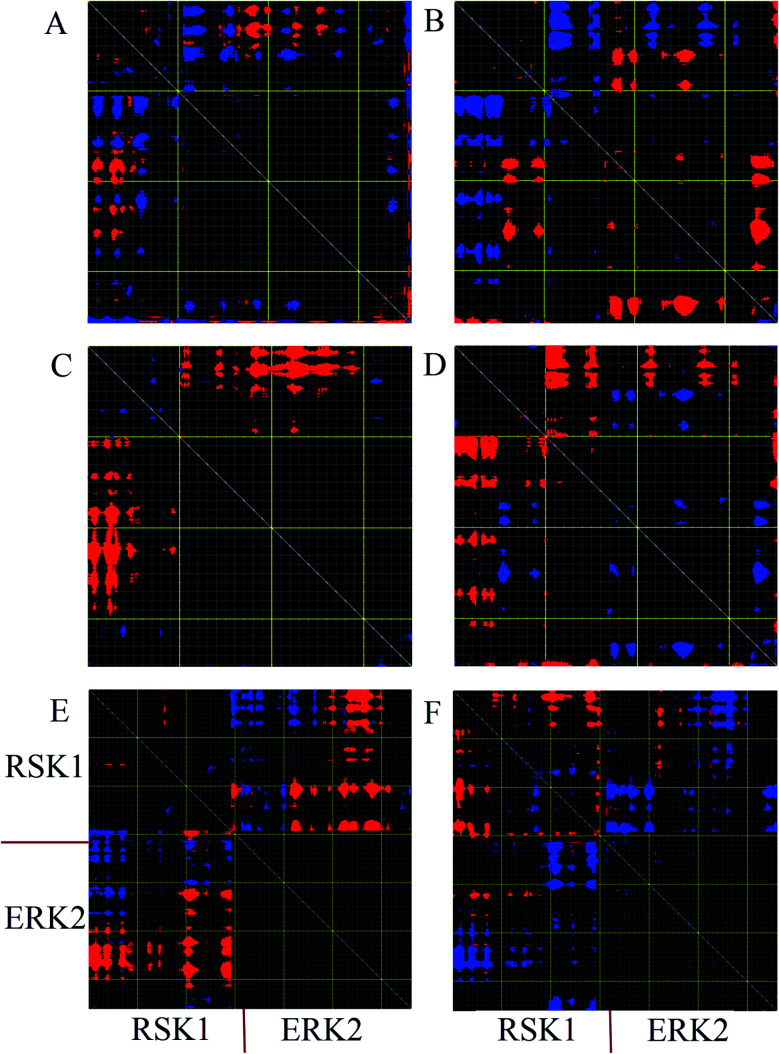
Distance fluctuation map (DFM) of active, inactive and complex forms of ERK2. Flexible blocks are colored for increase (blue) and decrease (red) of distance fluctuations. DFM of inactive-ERK2 in mode 9 (A) and mode 11 (B). DFM of active-ERK2 in mode 9 (C) and mode 11 (D). DFM of complex form in mode 7 (E) and mode 11 (F). Every pixel corresponds to a single residue. Grey lines were drawn every 10 residues, and yellow lines every 100 residues.

Distance fluctuation maps (DFM) were calculated to investigate variations of the Cα distances in the active, inactive, and complex forms of ERK2. According to DFM plots for modes 9 and 11, the distance between C-lobe and N-lobe was decreased in the active form, while this distance was increased in the inactive form. According to the DFM of mode 7 for complex form (Fig. 5E), the distance between the C-loop of RSK1 and the C-lobe of ERK2 was decreased and these parts interact with each other. However, in mode 11, the distance between RSK was decreased and RSK1 lost its interaction with ERK2.

## 4. Discussion

In the present study, we applied molecular dynamic simulation and normal mode analysis to evaluate the conformations of ERK2 in the forms of active (phosphorylated), inactive (unphosphorylated) and in the complex with RSK1 to determine its functional characteristics. MD simulation and normal mode analysis have been used as tools to investigate the local flexibility and large-scale conformational transitions. The results of the study show that ERK2 switches between open and closed forms during the simulation. In the active state, all critical regions of ERK2 were rearranged to be prepared for accepting the substrate and catalytic action. In contrast, all motions in the inactive-ERK2 lead to closure of the catalytic site. These findings were consistent with previous studies.^30–32^

According to our results (Fig. 1), the inactive ERK2 without ATP–Mg^2+^ (red graph) had a higher RMSD value and showed an unstable form compared to other structures. Whereas, active-ppERK2 (blue graph) in the absence of ATP (Fig. 1) demonstrated a more stable structure. Furthermore, the addition of ATP to active-ppERK2 resulted in remarkable structural motions. In other words, either active or inactive forms of ERK2 in the presence of ATP had higher structural motions, which led to the alteration of their conformations. As Bjarnadottir *et al.* and Johnson *et al.* stated, the closed conformation was observed when the ATP-binding site was occupied, Thr185 and Tyr187 residues were phosphorylated, and the substrate, which is finally phosphorylated by ERK2, was present.^31,33^

Similar to all protein kinases, ERK1/2 have two lobes, including a small N-terminal and a large C-terminal lobe. The most significantly functional regions in ERK2 are the glycine-rich loop, KIM, αC-helix, L16-helix, and activation segment. As the RMSF highlights, these regions had different flexibilities in the active and inactive forms in the presence and absence of ATP–Mg^2+^ (Fig. 2).^7^ Considering Rad_gyr_ graphs, as a character of structural compactness in proteins,^34^ the active states had more compact structures than the inactive states (Fig. S2†). As shown in Fig. 2, the open and closed conformations, related to the active and inactive forms of ERK2, were clearly observed. Structural investigations of ERK2 suggested that in the active-ppERK2, the N- and C-lobe tend to move closer to each other to be ready to interact with substrates. Instead, in the inactive state, the distance between C- and N-lobes was increased.

As Taylor *et al.* pointed out, the most marked region in the kinase core, in terms of sequence and length, is the activation segment, which acts as a switch to turn on and off the enzyme activity and to alter the conformation from closed to open in the catalytic cycle of ERK2.^35,36^ As Canagarajah *et al.* mentioned, controlling the enzyme activity is based on confirmation of the activation lip (P-Thr185 and P-Tyr187) in order to occupy the active site *via* covalently bound phosphates.^28^ Structural investigation of the activation segment demonstrated that in the active-ppERK2, this segment tends to move inside and to close to the N-lobe; while, in the inactive form, it tends to be close to the C-lobe. In other words, the active-ppERK2 has an open active site to recognize its substrates.^28^ Similarly, our study suggested that in the active-ppERK2 this segment moved towards the cavity of the enzyme and slightly approached the N-lobe. The activation loop was open and allowed the P + 1 loop to find the interaction site, whereas, in the inactive-ERK2, the P + 1 loop folded and decreased the accessibility of Thr187. This conformation blocked the phosphorylation and activation of ERK2's substrates because it impedes the P + 1 loop to find the interaction site (Fig. 3 and 4).^7,28,37^

Hanks *et al.* defined three conserved amino acids as Lys/Asp/Asp (K/D/D) that act as the catalytic core of protein kinases.^38^ Lys54 cooperates with Glu71 to produce a salt-bridge and Asp149, in a catalytic loop, traps the proton from the OH group from substrates and facilitates the nucleophilic attack of oxygen on the γ-phosphate of ATP.^39^ The second aspartate, Asp169, in the DFG motif, binds to Mg^2+^ ion to coordinate negative charges of phosphates groups from ATP.^7^ Given our findings, it was observed that the orientation of these residues changed during the simulation. Therefore, the distance between Lys54 and Glu71 declined and the salt-bridge was established (Fig. 3). In addition, both αC-helix and DFG-aspartate faced into the active site (Asp149 in the catalytic loop). In conclusion, the αC-helix-in and DFG-aspartate-in conformations, the distance graph (Fig. S3†), and accessible surface area for K/D/D motif (Fig. S4 and S5†) in the active-ppERK2 showed that the structure of ERK2 was more compact and was involved in the catalytic reaction.

Burkhard *et al.* have reported that ERK2 interacts with RSK1 through its DRS and FRS.^40^ Lee *et al.* employed hydrogen exchange mass spectrometry and single-point mutations in ERK2 to study the D-site and F-site docking domains. They suggested that in the active-ERK2, the FRS residues are located in the proximity of the activation loop and catalytic core.^41^ Based on our results from MD simulations, in the active-ppERK2 and ERK2–RSK1 complex, ED (T159, T160), CD (D318, D321), and hydrophobic groove (L115, L121, H125, Y128, L157) in DRS moved closer to each other during simulation; whereas, in the inactive-ERK2 these regions were far from each other. Furthermore, FRS is only accessible in the active-ERK2 that probably prepares ERK2 for the interaction with RSK1, while this region was covered by the activation loop in the inactive form (Fig. 4 and S7–S9†).

According to the above statements, active-ppERK2 with compact structure interacted through the D-site with the inactive-RSK1, where RSK1's activation loop faced the catalytic site of ERK2. As indicated previously, there are two important interfaces for the interaction of ERK2 with RSK1 including linear motif, which binds to the ERK2's docking groove, and Asp149 (catalytic loop) in ERK2 along with Thr573 (activation loop) in RSK1.^42^ It seems that at the beginning of the simulation, ERK2 was prepared to approach and interact with the RSK1. Then, in the middle of the simulation, the distance between Asp149 in ERK2 and Thr573 in RSK1 started increasing. In this situation, RSK1 properly turned to an active state and increased the distance from ERK2. Considering the catalytic cycle in kinases, the close and open conformations were constantly occurring in the MAPK pathway. Our findings from NMA (Fig. 5) suggested that the fluctuations and motions could be related to the catalytic reaction, which is due to the existence of ATP and substrates, such as RSK1, in kinases. In the presence of ATP in the structure, ERK2 triggered the catalytic reaction to activate downstream substrate (RSK1). In this way, the N-lobe and C-lobe of ERK2 stayed close to each other, while this orientation was not clearly observed in the absence of ATP.^43^

## 5. Conclusion

The MAPK signaling pathway includes several key protein kinases, which play a major role in cellular processes regulating cell growth, proliferation, differentiation, apoptosis, and migration. MAP kinases are specifically controlled in these activities such as their interactions with substrates and activating enzymes. In this study, we employed computational methods to investigate the structural and functional features of ERK2 in different forms such as active, inactive, and also in the complex with its downstream partner called RSK1. This survey showed how computational approaches could be practical for examining the biological behavior of ERK2 and helped us to find out the dynamics of this protein in its different forms. The results, with a focus on the dynamical properties of ERK2, confirmed the same behavior for ERK2 with other protein kinases. The salt-bridge, conformation of αC-helix and DFG, as well as open activation loop, are some features for distinguishing active and inactive kinases. According to the phosphorylation sites in the activation lip of ERK2, the catalytically active and inactive forms of the protein can be defined. When Thr185 and Tyr187 were phosphorylated by MEK1/2, known as the upstream protein of ERK2, the activation loop was open so that N- and C-lobes get close to each other. As a result, the salt-bridge was established between K54 and E71 in the N-lobe as these regions moved towards each other. In addition, the distances between K/D/D decreased, αC-helix and DFG faced into the active site (αC-helix-in and DFG-aspartate-in), and eventually, the entire structural conformations were ready to interact with downstream substrates including RSK1. Following the interaction of ERK2 with RSK1, *via* DRS in ERK2 and D-docking domain in RSK1, the catalytic reaction could be implemented. As ATP-binding site was taken up by ATP–Mg^2+^ in ppERK2, the protein kinase was catalytically active, and Asp149 located in the active site of ERK2 began to phosphorylate the Thr573 in the activation loop of RSK1.

In conclusion, the activation lip, as the critical regulator of the ERK2 activity, underwent conformational changes during the simulation. In the inactive structure, the activation lip was folded to block the P + 1 site and forced the neighboring structures, namely, KIM, the C-terminal extension, and helix C, into an open conformation. On the other hand, in a phosphorylated state, the lip was rearranged and exerted domain closure, resulting in the formation of the proline-directed P + 1 site. Moreover, the other changes occurred in L16, which caused close conformation as well as remodeling of phosphorylation-dependent interactions with other macromolecules. These changes in L16 also led to the formation of the ppERK2 dimer, which is physiologically important. In addition, phosphorylation of Tyr and Thr in the lip directly affected the lip's conformation in active and inactive ERK2. According to the previous studies and our findings, it is assumed that a specific conformation of activation lip in different ERK2's states contributes to the regulation of MAP kinase pathway and this region is conserved for activating point mutations in the enzyme.^28,44^

The ultimate goal of this study was to understand the function and dynamical behavior of ERK2 in complex with RSK1 in order to design inhibitory molecules to disrupt their interaction for blocking MAPK signaling pathway as to control cancer cell growth and proliferation as a potent and novel therapeutic method which has already been proposed in other studies for other cases.^45,46^

## List of abbreviations

Bcl-2: B-cell lymphoma 2
c-Myc: Proto-oncogene Myc
CREB: cAMP response element-binding protein
DFM: Distance fluctuation maps
DRS: D-recruitment site
eIF4B: Eukaryotic translation initiation factor 4B
ElNemo: Elastic network model
ERK2: Extracellular signal-regulated kinase 2
Ets1: ETS proto-oncogene 1
FRS: F-recruitment site
JNK: c-Jun N-terminal kinase
K/D/D: Lys/Asp/Asp
KIM: Kinase insert domain
MAPK: Mitogen-activated protein kinase
MD: Molecular dynamics
NF-κB: Nuclear factor kappa-light-chain-enhancer of activated B cells
PDB: Protein data bank
Rad_gyr_: Radius of gyration
RMSD: Root mean square deviation
RMSF: Root mean square fluctuation
RSK1: Ribosomal protein S6 kinase alpha-1
RTK: Receptor tyrosine kinase
TCF: Ternary complex factor
TIF1A: Transcription initiation factor 1A

## Conflicts of interest

There are no conflicts to declare.

## Supplementary Material

RA-011-D1RA01020D-s001
