# Supplementary material for: The functional regulatory details of ERK2 in complex with RSK1: an in silico insight
Source: RSC Adv. 2021 Mar 16;11(19):11048–56. doi: 10.1039/d1ra01020d (PMC8695832; doi:10.1039/d1ra01020d)
Supplement: RA-011-D1RA01020D-s001 [file RA-011-D1RA01020D-s001.pdf]

Electronic Supplementary Material (ESI) for RSC Advances.
This journal is © The Royal Society of Chemistry 2021

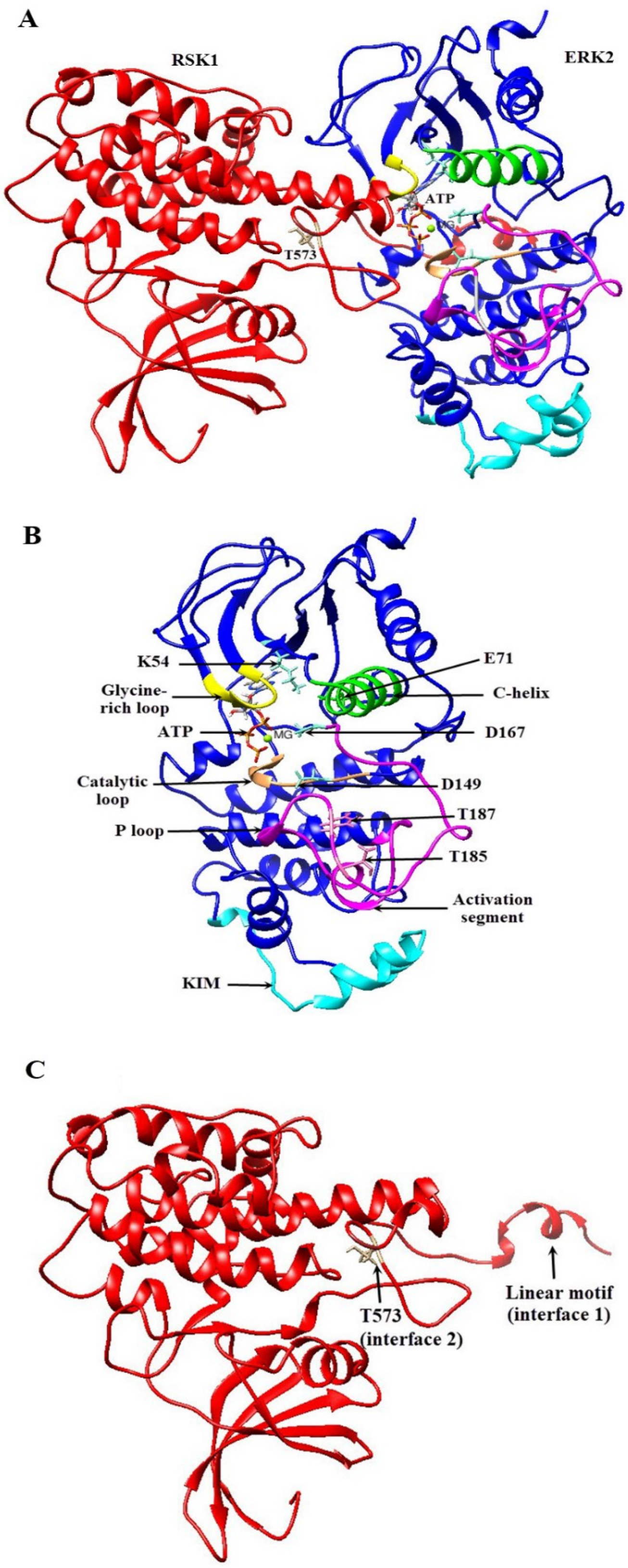

Figure S1: Critical regions in ERK2 and RSK1. (A) ERK2 in interaction with RSK1, (B) critical areas in ERK2 and (C) RSK1 structure with its interfaces.

Equation S1: Root-Mean-Square Deviation (RMSD)

$$RMSD = \sqrt{\frac{\sum_{i=0}^{N}(X_i - Y_i)^2}{N}}$$

Where: N is the number of atoms, m_i is the mas of atom i, X_i is he coordinate vector for target atom i, Y_i is the coordinate vector for reference atom i.

Equation S2: Root Mean Square Fluctuation (RMSF)

$$RMSF = \sqrt{\frac{1}{T}\sum_{t=1}^{T}\left|r_i(t) - \bar{r}_i\right|^2}$$

Where: T is the total number of frames, $r_i(t)$ is the position of ith atom at t^{th} frame and $\bar{r}_i$ is time-averaged position of ith atom

Equation S3: Radius of gyration (RG)

$$Rg = \sqrt{\frac{1}{N}\sum_{N}^{i=0}(r_i - r_m)^2}$$

Where: N is the number of atoms, r_i is atomic position and r_m is mean position of all atoms

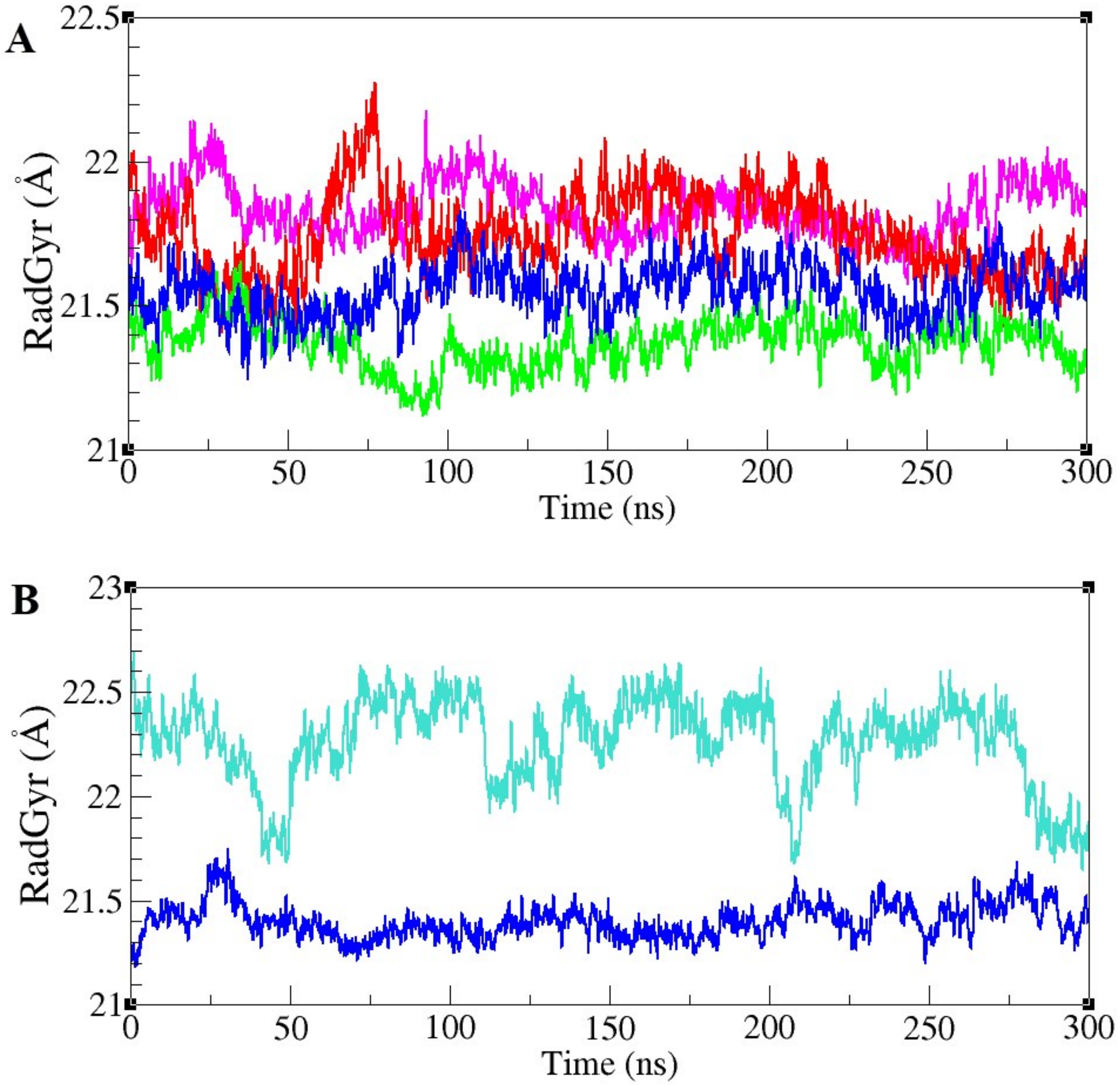

Figure S2. Radius of gyration (R_{gyr}) in ERK2 and ERK2-RSK1 complex during the simulation. (A) Rad_{gyr} values for inactive-ERK2 with ATP-Mg^{2+} (pink), inactive-ERK2 without ATP-Mg^{2+} (red), active-ppERK2 with ATP-Mg^{2+} (green), active-ppERK2 without ATP-Mg^{2+} (blue). The active-ppERK2 with ATP-Mg^{2+} (green) has the tightest structure. (B) Rad_{gyr} values for ERK2-RSK1 complex. The blue and turquoise lines represent Rad_{gyr} for ERK2 and RSK1, respectively.

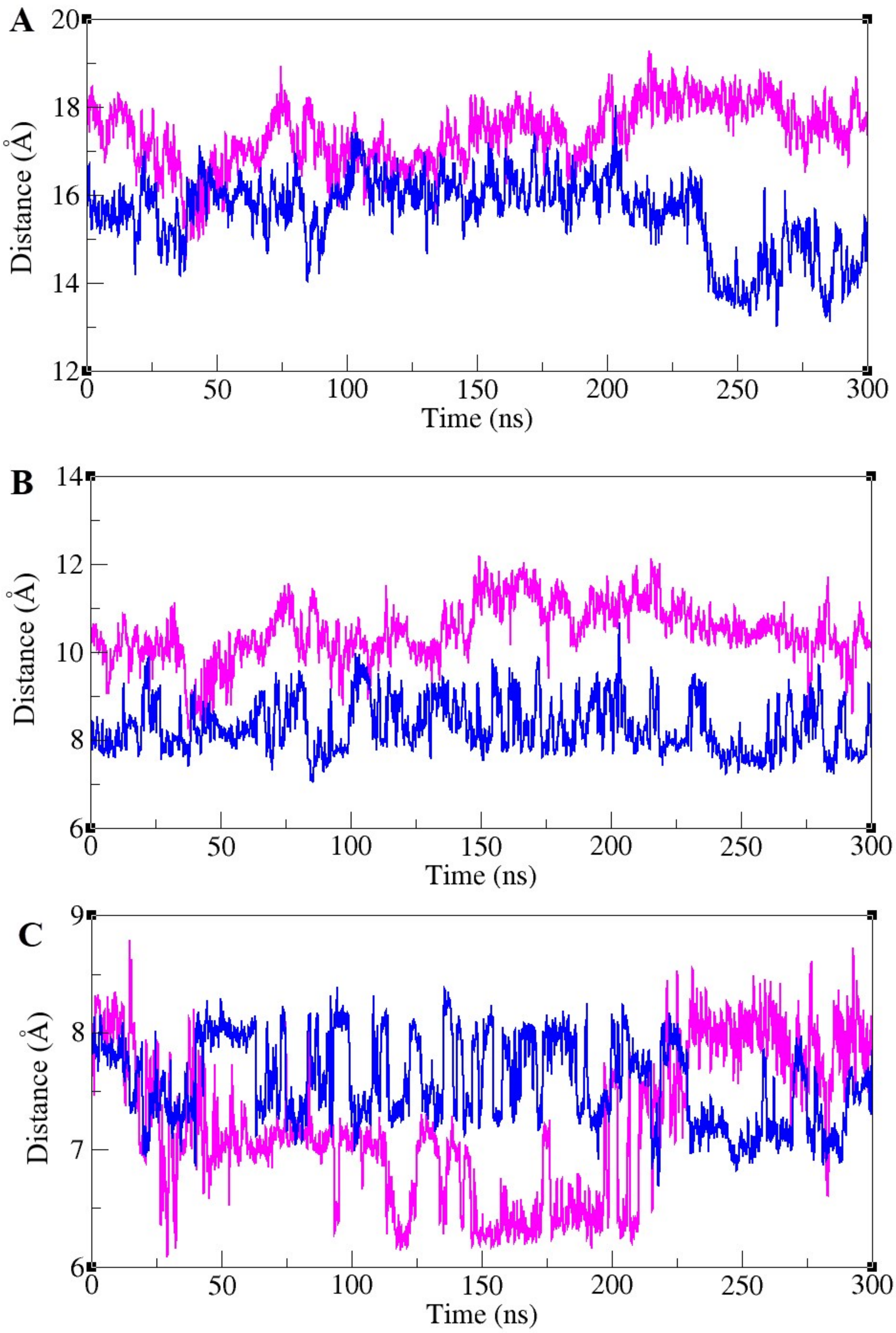

Figure S3. The distance graph for K/D/D motif (Lys54, Asp149, and Asp167) in inactive-ERK2 (pink) and active-ppERK2 (blue). (A) The distance between Lys54 and Asp149, (B) Lys54 and Asp167, and (C) Asp149 and Asp167.

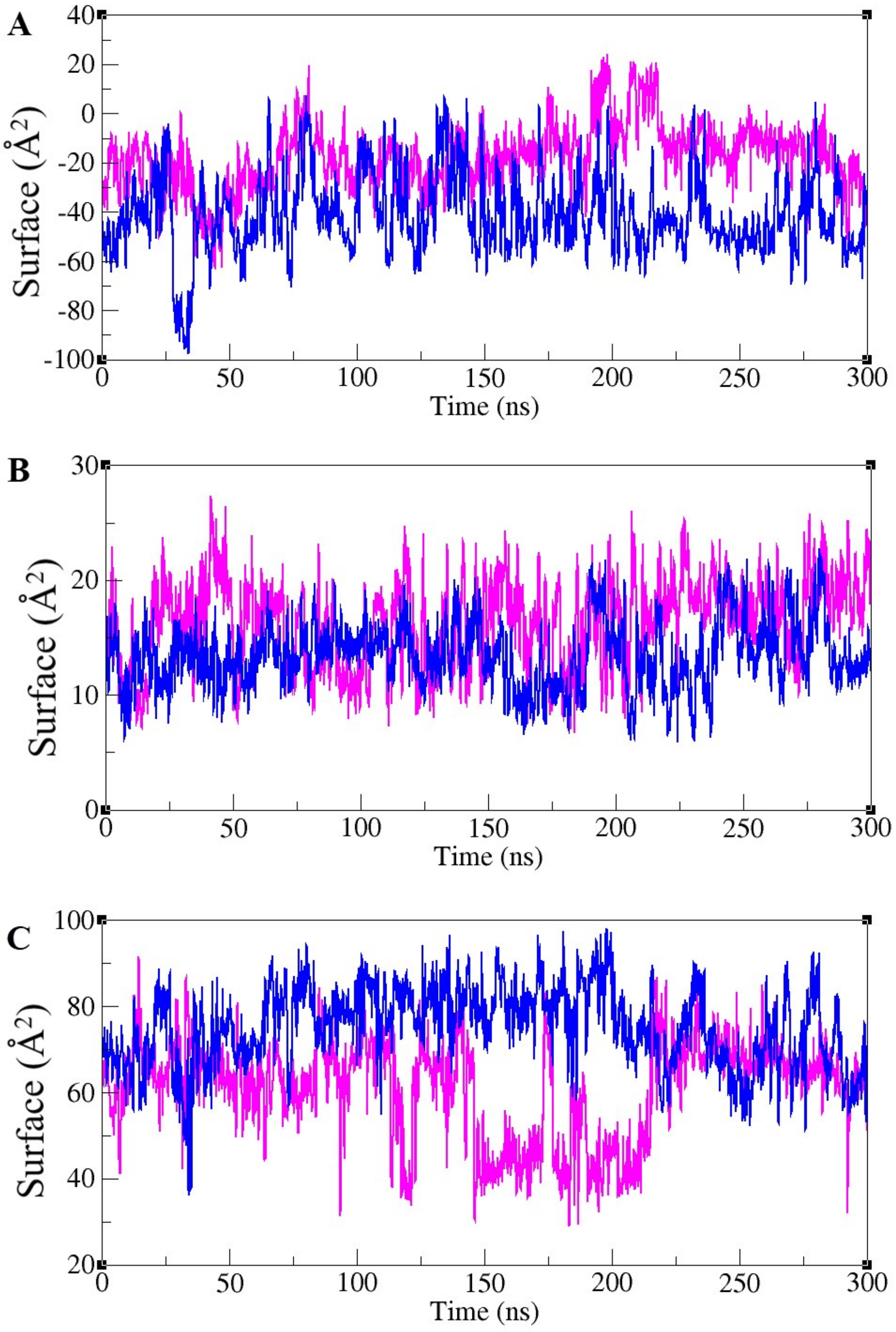

Figure S4. Accessible surface area for K/D/D motif (Lys54, Asp149, and Asp167) in two states of ERK2 during 300 ns simulation. Pink and blue lines represent inactive-ERK2 and active-ppERK2, respectively. (A) The accessible surface area of K54, (B) The accessible surface area of D149, and (C) The accessible surface area of D167.

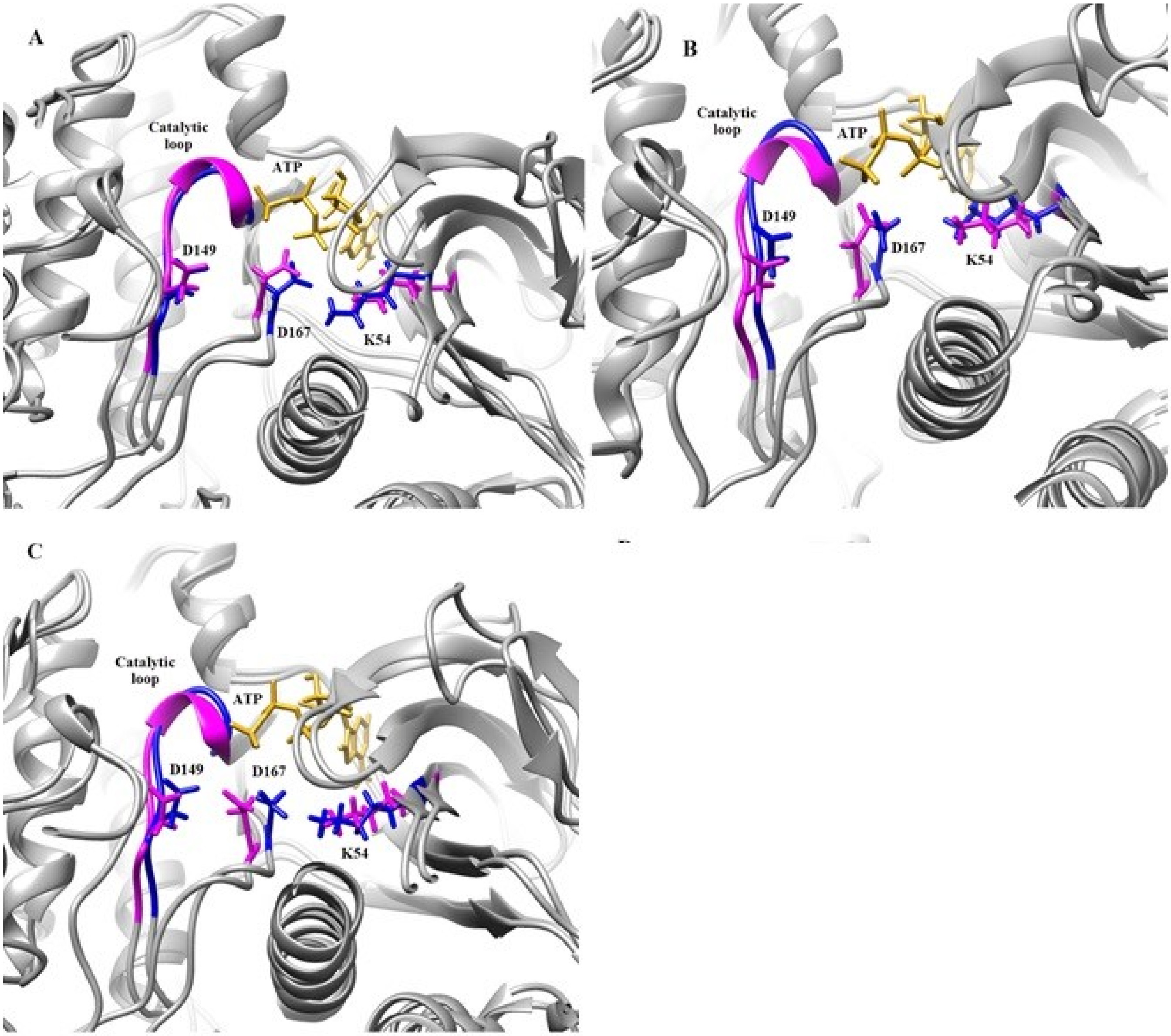

Figure S5. Structural movements in K/D/D (Lys/Asp/Asp) motif in active and inactive forms of ERK2 at different frames of simulation. The pink and blue regions represent inactive-ERK2 and active-ppERK2, respectively. (A) In active-ERK2 the relative orientation shows that the distance between K/D/D is less than that in inactive form. (B and C) In active form the distance is decreasing during the simulation in order to react with ATP.

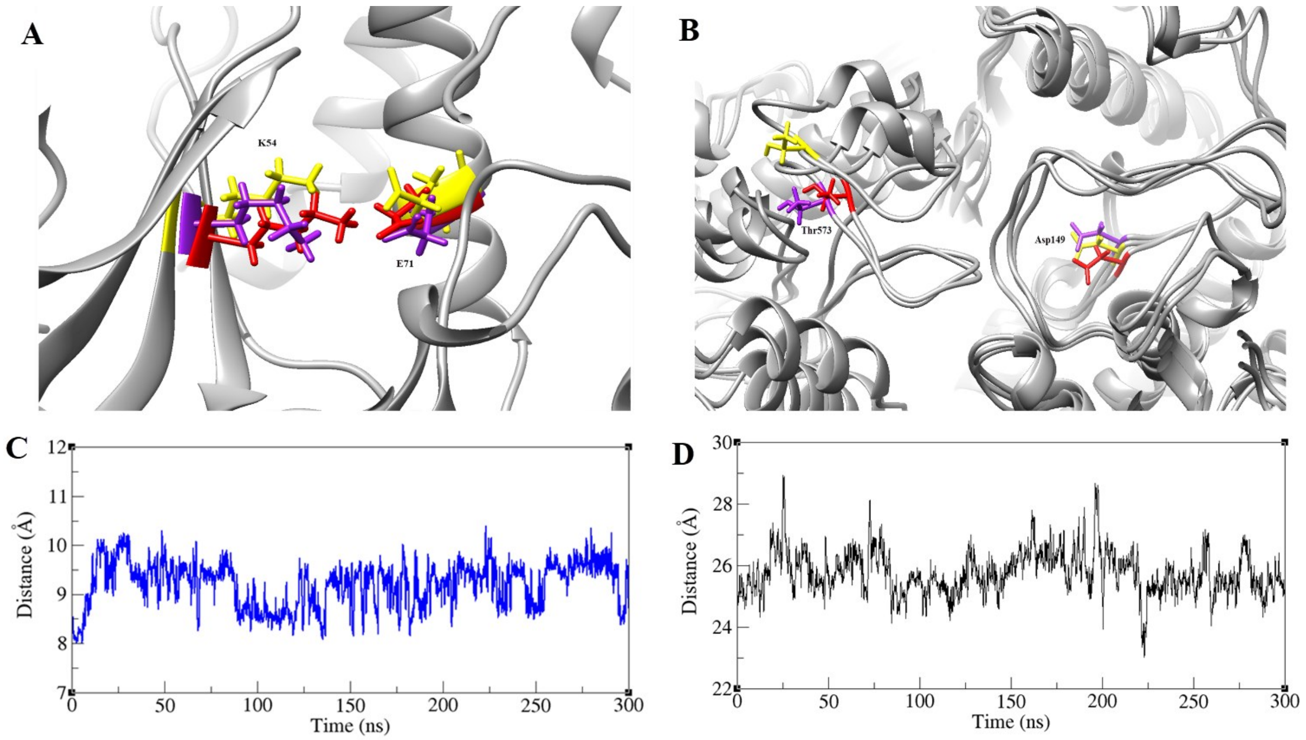

Figure S6. Distance between $Asp149_{ERK2}$-$Thr573_{RSK1}$ and $Lys54_{ERK2}$-$Glu71_{RSK1}$ in ERK2-RSK1 complex. The orientation of Lys54 and Glu71 (A), Asp149 and Thr573 (B) at initial point (red), middle (yellow), and end of simulation (pink). (C and D) Distance graph for Lys54 and Glu71 and Asp149 and Thr573.

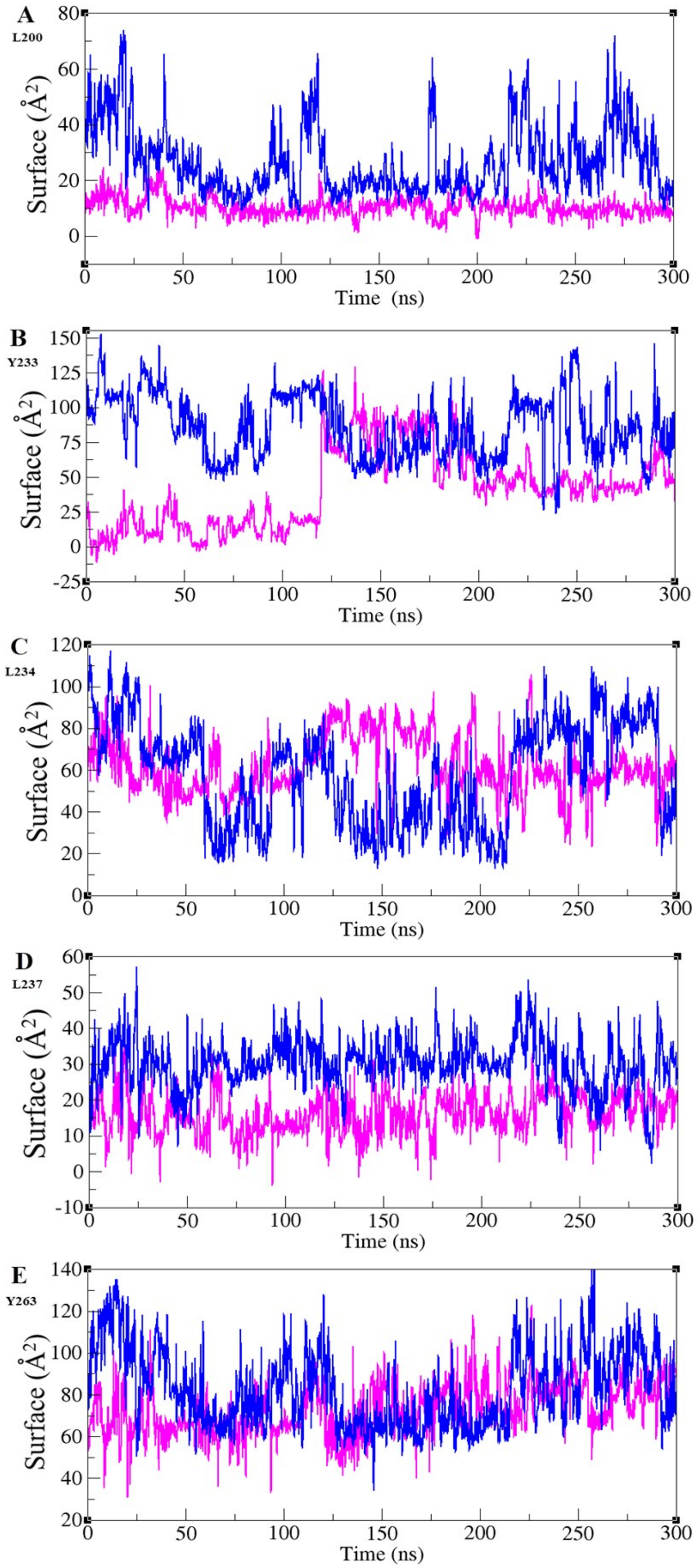

Figure S7. Accessible surface areas of F-recruitment site (FRS) that interacts with the D-docking Domain in ERK2's substrates. Inactive-ERK2 and active-ppERK2 are depicted in pink and blue, respectively. Generally, the surface area of this site in active-ppERK2 is more accessible than inactive form.

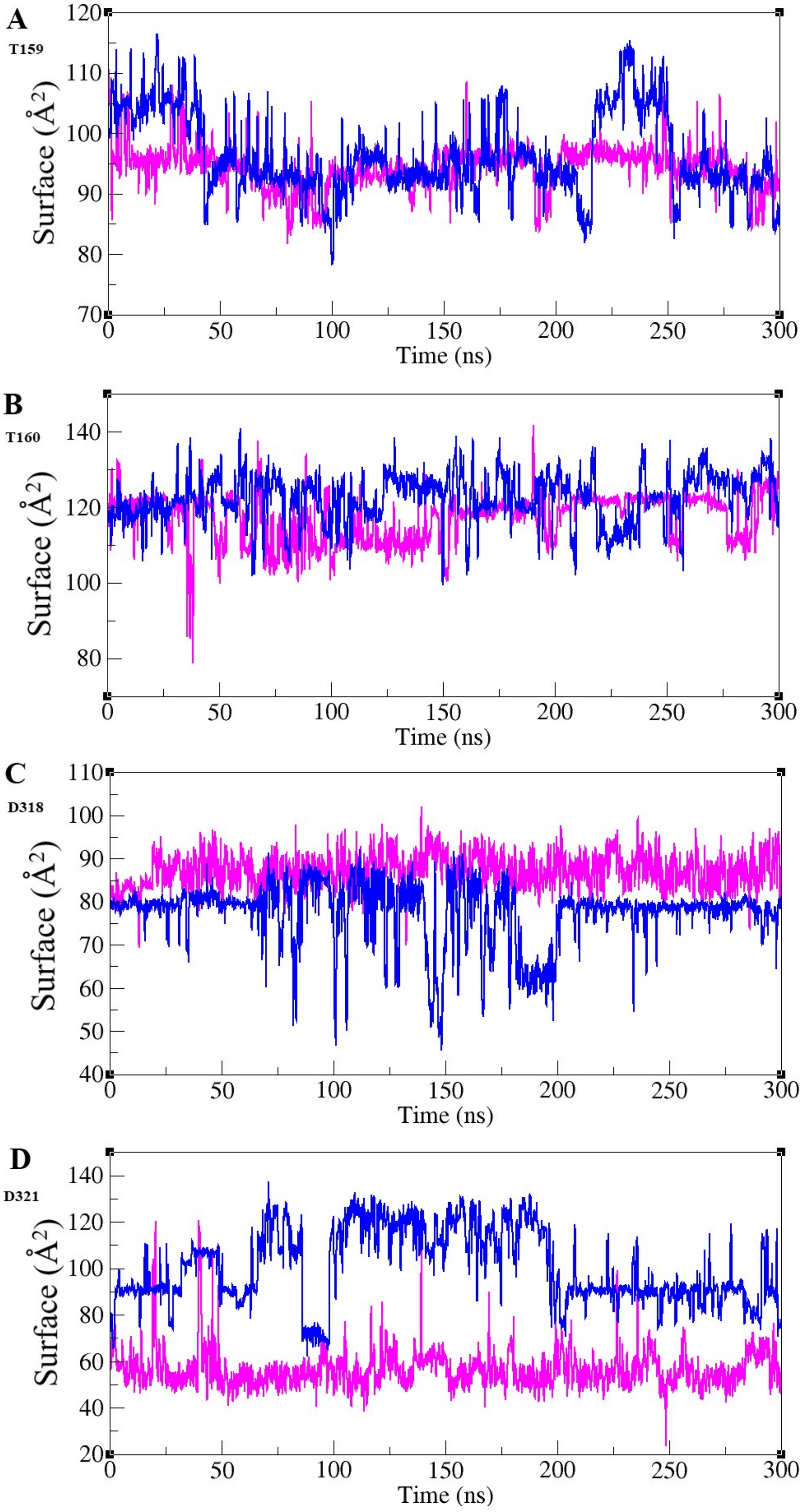

Figure S8. Accessible surface areas of ED and CD in D-recruitment site (DRS) that interacts with the D-docking Domain in ERK2's substrates. A and B represent ED site (Thr159-Thr160), C and D represent CD site (Asp318-Asp321). Inactive-ERK2 and active-ppERK2 are depicted in pink and blue, respectively. Generally, the surface area of this site in active-ppERK2 is more accessible than inactive form (except for Thr318).

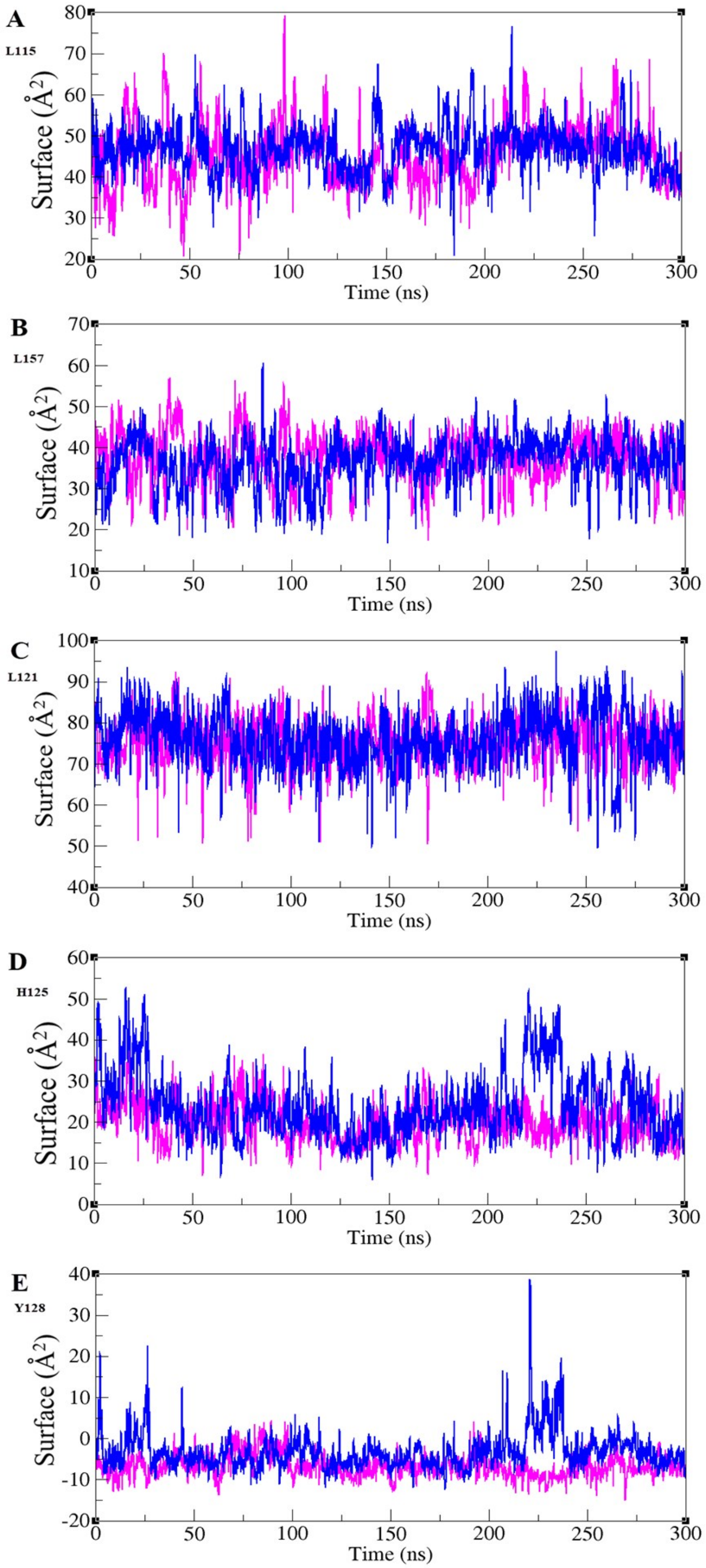

Figure S9. Accessible surface area of Hydrophobic groove in D-recruitment site (DRS) that interacts with the D-docking Domain in ERK2's substrates. Inactive-ERK2 and active-ppERK2 forms are depicted in pink and blue, respectively.
